# Simulation of Arrhythmogenic Effect of Rogue RyRs in Failing Heart by Using a Coupled Model

**DOI:** 10.1155/2012/183978

**Published:** 2012-09-29

**Authors:** Luyao Lu, Ling Xia, Xiuwei Zhu

**Affiliations:** ^1^Department of Biomedical Engineering, Wenzhou Medical College, Wenzhou 325035, China; ^2^Department of Biomedical Engineering, Zhejiang University, Hangzhou 310027, China

## Abstract

Cardiac cells with heart failure are usually characterized by impairment of Ca^2+^ handling with smaller SR Ca^2+^ store and high risk of triggered activities. In this study, we developed a coupled model by integrating the spatiotemporal Ca^2+^ reaction-diffusion system into the cellular electrophysiological model. With the coupled model, the subcellular Ca^2+^ dynamics and global cellular electrophysiology could be simultaneously traced. The proposed coupled model was then applied to study the effects of rogue RyRs on Ca^2+^ cycling and membrane potential in failing heart. The simulation results suggested that, in the presence of rogue RyRs, Ca^2+^ dynamics is unstable and Ca^2+^ waves are prone to be initiated spontaneously. These release events would elevate the membrane potential substantially which might induce delayed afterdepolarizations or triggered action potentials. Moreover, the variation of membrane potential depolarization is indicated to be dependent on the distribution density of rogue RyR channels. This study provides a new possible arrhythmogenic mechanism for heart failure from subcellular to cellular level.

## 1. Introduction

Calcium is considered to be the key ion in mediating the process of cardiac excitation-contraction coupling (E-C coupling). Since the discovery of Ca^2+^ sparks in 1993 [1], Ca^2+^ sparks have been widely accepted to be the stereotyped elementary Ca^2+^ release events in the intact myocyte. Sparks arise via clusters of ryanodine receptors (RyRs) localized in the junctional SR (jSR) which is in close apposition to transverse tubules (TTs) [2]. In a diastolic myocyte, spontaneous Ca^2+^ sparks occur randomly at very low frequency, even in the absence of Ca^2+^ influx. During a single muscle twitch, Ca^2+^ influx via sarcolemmal L-type Ca^2+^ channels will trigger synchronously occurrence of thousands of sparks, summation of which in space and time causes a global steep rise of Ca^2+^ concentration named Ca^2+^ transient. However under some pathological conditions, successive recruitment of Ca^2+^ sparks tends to evolve into Ca^2+^ waves propagating across the myocytes which might trigger ventricular arrhythmias [3].

With the improvement of optical methods and innovative techniques, microscopic Ca^2+^ signals at the subcellular level have been extensively investigated and characterized. In addition to Ca^2+^ sparks via clustered RyRs, nonspark Ca^2+^ release events, named Ca quarks, activated by low-intensity photolysis of Ca^2+^-caged compounds [4] or by inward Na^+^ current, *I*_Na_ [5], could elicit spatially homogeneous but small Ca^2+^ transient. These quarks are likely to be mediated via one or a few RyR channels called rogue RyRs [6]. Differing from RyR clusters that underly sparks, rogue RyRs are thought to be uncoupled with each other and behave in ways more like the characteristic of single RyR channels [7]. Although detection of these small rogue RyR channels is difficult by conventional instruments, some researchers have suggested that, besides sparks, the nonspark pathway via rogue RyRs explains a part of SR Ca^2+^ leak [8, 9]. Quantitatively, with the optical superresolution technique, Baddeley et al. have indicated that there are greater numbers of rogue RyR groups than large RyR clusters [10]. An experimental study that Ca^2+^ waves are inhibited without affecting Ca^2+^ sparks by ruthenium red suggests a nonspark producing RyR channels which are important to propagation of Ca^2+^ wave [11]. Direct visualization of small local release events has been made possible by recent technical innovations. Brochet et al. claimed that they have directly visualized quark-like or “quarky” Ca^2+^ release events which might depend on the opening of rogue RyRs (or small cluster of RyRs) in rabbit ventricular myocytes [12].

SR Ca^2+^ leak consists of two components: RyR-dependent leak and RyR-independent leak [8]. The former is thought to be comprised of spark-mediated leak (visible leak) and non-spark-mediated leak (invisible leak). Elevated SR Ca^2+^ leak would contribute to delayed afterdepolarizations (DADs) and consequently arrhythmia in heart failure (HF) [13]. Besides spark-mediated leak, additional Ca^2+^ leak via rogue RyRs may be an important factor in disturbing Ca^2+^ dynamics and triggering Ca^2+^ waves [11, 14]. However, how do these abnormal Ca^2+^ release events affect cellular electrophysiological properties? The precise relationships between property of rogue RyRs and Ca^2+^ handling as well as cellular electrophysiology in failing heart are not completely clear.

In this paper, we developed a coupled mathematical model including Ca^2+^ cycling processes from subcellular to cellular level and electrophysiology of the ventricular myocyte. The proposed coupled model was then applied to study the effects of Ca^2+^ release via rogue RyRs on subcellular spatiotemporal Ca^2+^ cycling and on the possible membrane potential changes in failing heart.

## 2. Methods

Subcellular Ca^2+^ release events and cellular Ca^2+^ cycling as well as corresponding membrane potential were simulated synchronously by a coupled model. The model consists of two parts: a two-dimensional (2D) spatial Ca^2+^ reaction-diffusion model and an electrophysiological model of the ventricular myocyte.

### 2.1. A Subcellular Ca^2+^ Reaction-Diffusion Model

**Figure 1:**
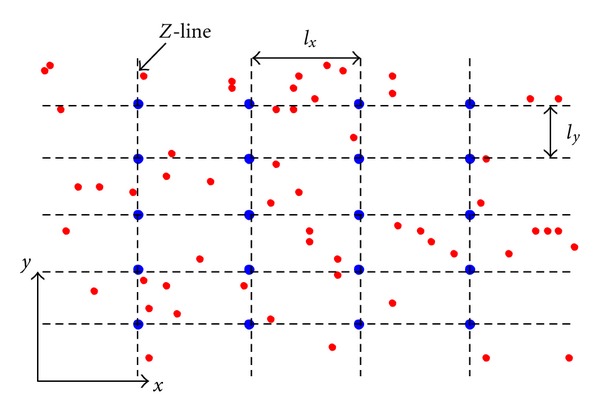
Geometry of RyRs distribution. The blue dots represent Ca^2+^ release units consisting of clusters of RyRs. *l*_*x*_ = 2.0 *μ*m and *l*_*y*_ = 1.0 *μ*m. The small red dots are rogue RyRs which scatter over the plane randomly. In this figure, *N*_rogue_ is equivalent to 1.0 rogue RyR/*μ*m^2^.

The shape of the cardiac myocyte in the model is represented as a circular cylinder 100 *μ*m in length and 10 *μ*m in radius. However, because of quasi-isotropic diffusion of Ca^2+^ on the transverse section [15], a 2D model was used in our simulation work (Figure 1), where *x* axis denotes the cell's longitudinal direction and *y* axis is along the *Z*-line. It could still describe most of the key properties of Ca^2+^ waves, but needs much less computation work than a 3D model. The 2D spatiotemporal Ca^2+^ reaction-diffusion model is described based on a reaction-diffusion system proposed by Izu et al. [16]. Figure 1 shows the subcellular structural representation of RyRs network. The *x*-axis denotes the cell's longitudinal direction and the *y*-axis is along the *Z*-line. The blue dots represent RyR clusters which account for Ca^2+^ sparks. The small red dots are the rogue RyR channels which raise Ca^2+^ quarks. Rogue RyRs are distributed in a stochastic manner. *N*_rogue_ is referred to the distributing density of rogue RyRs with the unit of rogue RyR/*μ*m^2^.

The free Ca^2+^ concentration [Ca^2+^]_*i*_ in the reaction-diffusion is described by a differential equation as follows:

(1) ∂[Ca2+]i∂t=Dx∂2[Ca2+]i∂x2+Dy∂2[Ca2+]i∂y2+Jdye+Jbuffers+Jpump+Jleak+Jsub-rel,

where *D*_*x*_ and *D*_*y*_ are the diffusion coefficients; *J*_dye_ and *J*_buffers_ are due to fluorescent indicator dye and endogenous Ca^2+^ buffer, respectively; *J*_pump_ is pumping rate of SR Ca^2+^ ATPase; *J*_leak_ is defined as a RyR-independent leak flux which is small and invisible and persists in the presence of RyR inhibition [8]; *J*_sub-rel_ is summation of Ca^2+^ release fluxes in the 2D subcellular model which consists of two types of Ca flows as follows:

(2) Jsub-rel=∑i,jJcluster(xi,yj)+∑m,nJrogue(xm,yn),Jcluster(xi,yj)=Vcluster([Ca2+]SR−[Ca2+]i,(xi,yj)),

where *J*_cluster_(*x*_*i*_, *y*_*j*_) is Ca^2+^ release flux via a cluster of RyRs located on (*x*_*i*_, *y*_*j*_), *V*_cluster_ is maximal *J*_cluster_ conductance equivalent to 1.97 × 10^−8^ ms^−1^, and *J*_rogue_(*x*_*m*_, *y*_*n*_) is Ca^2+^ release flux via a rogue RyR channel located on (*x*_*m*_, *y*_*n*_), equivalent to 3.3166 × 10^−9^ pmol/ms.

Firings of the two types of RyR channels are considered to be stochastic processes and treated by the Monte Carlo simulation in our work. To evaluate the effects of SR luminal Ca^2+^ concentration ([Ca^2+^]_SR_) on SR Ca^2+^ release, we integrate a new parameter *k*_CaSR_ into the probability of firing of Ca^2+^ sparks or quarks (*P*_*j*_, *j* = *cluster* for RyR clusters, and *j *= *rogue* for rogue RyRs) as follows:

(3) kCaSR=kmax⁡1+(DSR/[Ca2+]SR)nSR,Pj=Pmax⁡1+(Kmj/[Ca2+]i)njkCaSR,

where *k*_max⁡_ = 2.0, the Hill coefficient *n*_SR_ = 4.5, *P*_max⁡_ = 0.3/event/ms, *n*_cluster_ = 1.6, and *n*_rogue_ = 1.0 for the less coupled gating of rogue RyRs than RyR clusters. *D*_SR_ is luminal Ca^2+^ sensitivity parameter of Ca^2+^ release events, and *K*_*mj*_ is cytoplasmic Ca^2+^ sensitivity parameter of RyR clusters or rogue RyR channels. In our simulation work, *K*_*m*rogue_ was always set to be of the same value as *K*_*m*cluster_; thus *K*_*m*_ was used to represent the value of *K*_*m*cluster_ and *K*_*m*rogue_.

In this study, the simulation of subcellular Ca^2+^ handling was performed on the longitudinal section of a cardiac myocyte with the size of 100 *μ*m × 20 *μ*m along the cellular longitudinal direction (*x*-axis) and *Z*-line (*y*-axis), respectively. The number of RyR clusters was 49 × 19 along *x* and *y* axes, respectively, and the total number of rogue RyRs was *N*_rogue_ × 2000 *μ*m^2^. The diffusion partial differential equation was approximated by the finite difference method (FDM) with a time-step size of 0.01 ms and a mesh size of 0.1 *μ*m.

Because of the stochasticity of opening of RyR clusters and rogue RyRs, the properties of Ca^2+^ signalling were described by statistical results by carrying out repeated Monte Carlo simulations. All averaged data were expressed as mean ± SEM. One-way analysis of variance (ANOVA) was used for comparison and *P* < 0.05 was taken to indicate statistical significance.

### 2.2. A Cellular Electrophysiological Model

The electrophysiological behavior of a myocardial cell is modelled based on a cardiac action potential model proposed by Ten Tusscher and Panfilov [17]. The voltage across the cell membrane can be described with the following differential equation:

(4) dVmdt=−1Cm(INa+ICaL+Ito+IKr+IKs+IK1+INaK+INaCa+IpCa+IpK+IbCa+IbNa+Istim),

where *C*_*m*_ is the membrane capacitance, *I*_stim_ is a stimulus current, and *I*_*x*_ denotes all kinds of ionic currents across the sarcolemma. 

However, different from the Ca^2+^ dynamical system by Ten Tusscher et al., global SR Ca^2+^ release current *J*_*rel*⁡_ at the cellular level is calculated by the summation of local Ca^2+^ release fluxes in the 2D subcellular model:

(5) Jrel⁡=krel⁡Jsub-rel=krel⁡(∑i,jJcluster(xi,yj)+∑m,nJrogue(xm,yn)),

where *k*_*rel*⁡_ is a constant multiplier equivalent to 22.25 in our coupled model.

### 2.3. Heart Failure Model

Changes of Ca^2+^ cycling as well as other ionic currents have been observed in failing heart; thus we modified the parameters of our coupled model to mimic abnormal Ca^2+^ dynamics and electrophysiological properties in heart failure from subcellular to cellular levels.

#### 2.3.1. Ca^2+^ Handling

**(a) SR *Ca* ^2+^ Release Channels**

In HF, RyR channels would become unstable due to phosphorylation of protein kinase A (PKA) [18] or Ca^2+^/calmodulin-dependent-protein-kinase-I- (CaMKI-) induced hyperphosphorylation [19] and be oversensitive to cytoplasmic Ca^2+^ and SR luminal Ca^2+^ [20]. In our simulation study, *K*_*m*_ was set to be 7.5 *μ*M and *D*_SR_ was 2.5 mM under the condition of heart failure, while 15 *μ*M and 3.25 mM, respectively, under control condition.

**(b) SR *Ca* ^2+^ Pump**

Pumping activity of SR Ca^2+^ ATPase in failing heart is reduced as shown in experimental studies [21]. A 45% reduction in *J*_pump_ of a failing myocyte is incorporated into our HF model.

**(c) SR *Ca* ^2+^ Leak**

Spontaneous openings of RyR clusters and rogue RyRs at diastole are the main contributors to SR Ca^2+^ leak as the form of Ca^2+^ sparks and quarks. Because of instability of RyR channels, RyR-mediated Ca^2+^ leak from SR increased in the resting HF myocyte. However, RyR-independent leak was unaltered in our HF model.

#### 2.3.2. Ionic Current across the Sarcolemma

**(a) Inward Rectifier Potassium Current: *I*_*K*1_**

In heart failure, *I*_K1_ was shown to be reduced in many studies [22, 23]. In our HF model the current density of *I*_K1_ was assumed to decrease by 20%.

**(b) Slowly Activated Delayed Rectifier Potassium Current: *I*_*Ks*_**

*I*_Ks_ is the slowly activated component of delayed rectifier potassium current. In the failing canine hearts *I*_Ks_ has been shown to be downregulated by nearly a half [24]. Therefore, maximal *I*_Ks_ conduction was changed to 50% of the value used in nonfailing myocytes.

**(c) Transient Outward Potassium Current: *I*_*to*_**

According to an experimental result, the current density of *I*_to_ in HF declined to 64% of the value in control cardiac cells [25], so that in our simulations *I*_to_ was reduced to 64% in failing myocytes.

**(d) Fast Na Current: *I*_*Na*_**

It has been reported that the peak density of *I*_Na_ decreased significantly in heart failure [26]. Therefore, the maximal *I*_Na_ conductance *G*_Na_ was set to be 8.902 nS/pF in the failing myocytes, while equivalent to 14.838 nS/pF in the nonfailing myocytes.

**(e) Na-Ca Changer Current: *I*_*NaCa*_**

The activity and/or gene expression of Na/Ca changer was found to be increased obviously in many experiments [27, 28]. Thus we upregulated *I*_NaCa_ by 65% in the failing myocytes.

**(f) Na-K Pump Current: *I*_*NaK*_**

As shown in the experimental study, the concentration of Na/K ATPase in the failing heart was reduced by 42% [29], so that reduction of *I*_NaK_ by the same proportion was incorporated in our HF model.

**(g) Ca Background Current: *I*_*bCa*_**

Inward *I*_bCa_ was considered to balance Ca^2+^ extrusion via Na/Ca exchanger and sarcolemmal Ca^2+^ pump at resting potential. In our HF model the conductance of *I*_bCa_ was increased due to the increase of *I*_NaCa_.

The different values of parameters in nonfailing and failing myocyte models are shown in Table 1. 

## 3. Results

### 3.1. Ca^2+^ Cycling and *V*_*m*_ in HF

**Figure 2:**
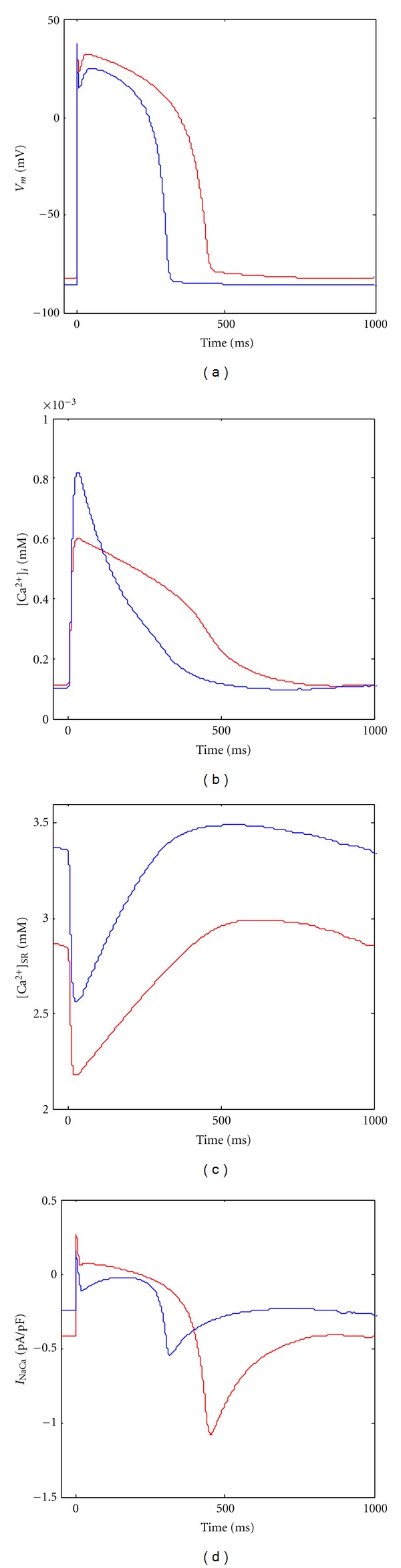
Simulation results of membrane potential (*V*_*m*_) (a), cytoplasmic Ca^2+^ concentration ([Ca^2+^]_*i*_) (b), SR luminal Ca^2+^ concentration ([Ca^2+^]_SR_) (c), and *I*_NaCa_ (d) during a single twitch. The red curve represents the time course of those parameters in failing myocytes, while the blue curve is the time course of those parameters in control myocytes.

With the proposed coupled model, firstly we simulated the action potential and calcium cycling by applying a stimulus with a frequency of 1 Hz, duration of 1 ms, and an amplitude of 7 pA.  Figure 2 shows the simulation results of membrane potential, cytoplasmic Ca^2+^ concentration, Ca^2+^ concentration in SR lumina, and the Na^+^/Ca^2+^ exchanger current after 10th stimulus. While blue curves in Figure 2 are obtained under the physiological conditions, red curves are under the pathological conditions, that is, heart failure. Compared with that in nonfailing myocytes, the plateau of action potential (AP) shows a larger amplitude and longer duration, causing a significant increase in AP duration (~45% longer than in normal condition) in heart failure. Meanwhile, decrease of the maximal conductivity of *I*_K1_ in heart failure makes the resting potential elevate 2~3 mV. However, the amplitude of AP overshot is smaller in heart failure, which is due to the reduction of fast inward current *I*_Na_. Then at the early stage of rapid repolarization, a weakened notch is observed in heart failure AP, which is caused by a decrease of *I*_to_. Moreover, the prolonged plateau is mainly due to decease of maximal conductivity of *I*_Ks_.

 For the calcium handing in heart failure, it is mainly characterized by a significant impair of global Ca^2+^ transient and a much slower decay of calcium concentration. Moreover, the SR Ca^2+^ store is smaller in heart failure, that is, [Ca^2+^]_SR_ is ~15% lower in resting cells, and the restoring rate of SR calcium is slower than that on control condition. Due to the changes of AP morphology and calcium transient curves together with increase of the activity of Na/Ca exchanger, the curve of *I*_NaCa_ in heart failure differs significantly from that under normal condition. This can be seen in Figure 2(d); that is, in heart failure, both the inward and outward currents of *I*_NaCa_ are increased. However, it takes a longer time to reach the peak of inward current, and the amplitude of inward *I*_NaCa_ in resting stage is also larger compared with that under control conditions.

### 3.2. Dependence of DAD on Rogue RyR

**Figure 3:**
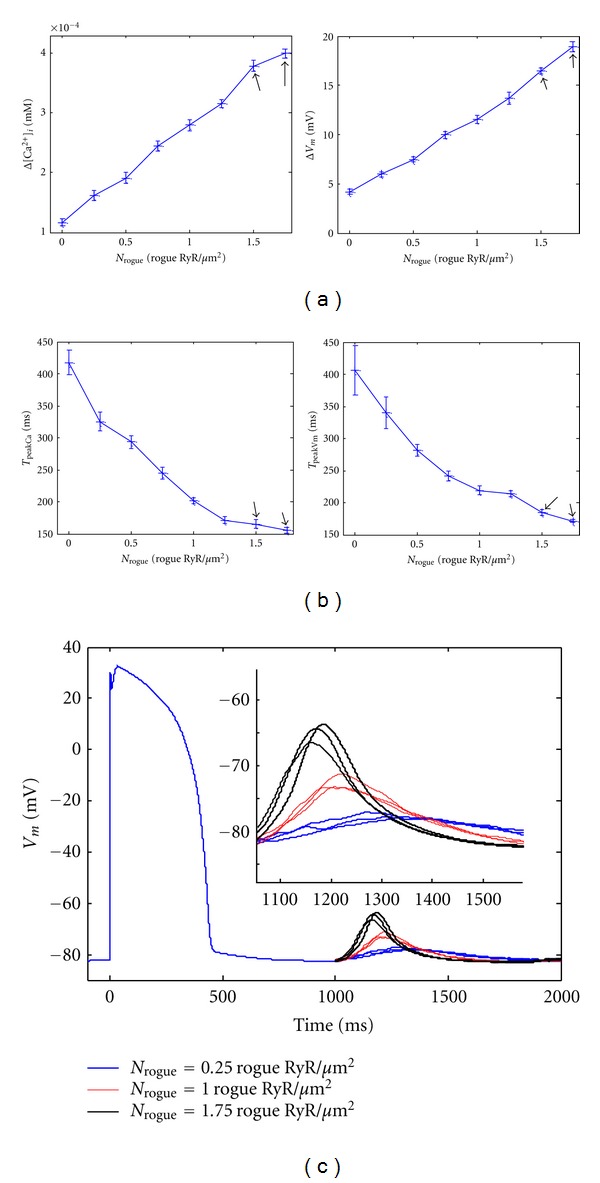
(a) Effects of distribution density of rogue RyRs on global change in [Ca^2+^]_*i*_ (Δ[Ca^2+^]_*i*_, left image) and depolarization of membrane potential (Δ*V*_*m*_, right image ) without any external stimulus in heart failure. (b) Dependence of *T*_peakCa_ and *T*_peak*V*_*m*__ on *N*_rogue_. *T*_peakCa_ represents the time to reach the peak of global [Ca^2+^]_*i*_ from the end of resting stage, and *T*_peak*V*_*m*__ is the time to reach the peak of *V*_*m*_. For each *N*_rogue_, we simulated 10 times under the same conditions to get statistical results due to the randomness of opening of RyR channels. The values denoted by arrows in (a) and (b) are recorded when *I*_Na_ = 0 pA. (c) Three groups of simulation results of *V*_*m*_ when *N*_rogue_ = 0.25, 1.0, and 1.75 rogue RyR/*μ*m^2^ (blue, red, and black curves, resp.). The inset is the enlarged view of depolarization of membrane potential during the period of 1100 ms ~ 1500 ms. *I*_Na_ is also set to be 0 pA when *N*_rogue_ = 1.75 rogue RyR/*μ*m^2^.

In heart failure myocytes, the RyR channels become very unstable and are more likely to open with the same values of [Ca^2+^]_*i*_ and [Ca^2+^]_SR_ as those under normal conditions. However, the calcium release current through a RyR cluster decreases as the SR calcium store is partly unloaded, which is observed as reduction of amplitude and area of calcium sparks. Indeed, the simulation results by using our coupled model show that, although more spontaneous calcium sparks occur in failing myocytes, propagating Ca^2+^ waves are seldom found when there is no rogue RyRs on the 2D subcellular space without any stimulus. These spontaneous calcium sparks would slightly elevate global [Ca^2+^]_*i*_ on the cellular level (Δ[Ca^2+^]_*i*_ = (1.16 ± 0.06) × 10^−4^ mM, *n* = 10) and depolarize transmembrane potential with a tiny amplitude (Δ*V*_*m*_ = 4.21 ± 0.23 mV, *n* = 10) as shown in Figure 3(a). 

However, how do Ca^2+^ dynamics and electrophysiological properties of a failing myocyte change in the presence of rogue RyR channels? We integrate rogue RyR channels into the 2D RyR grid and vary their distribution density *N*_rogue_ to investigate the precise effect of rogue RyR channels on Ca^2+^ handling and membrane potential. When the density of rogue RyRs is relatively low, for example, *N*_rogue_ = 0.25 rogue RyR/*μ*m^2^, spontaneously occurring calcium sparks are frequently observed in the subcellular region of HF myocytes during the resting state. Occasionally Ca^2+^ waves are formed, albeit at a small area, by recruiting several adjacent Ca^2+^ sparks. Those small Ca^2+^ waves could not propagate across the whole myocyte, but self-abort during a short time. Similar to the condition without rogue RyRs, global [Ca^2+^]_*i*_ and membrane potential are not affected severely by those Ca^2+^ release events under the condition with low density of rogue RyRs as shown in Figure 3(a) (Δ  [Ca^2+^]_*i*_ = (1.61 ± 0.08)× 10^−4^ mM, Δ*V*_*m*_ = 5.95 ± 0.29 mV, *n* = 10). 

Amplification and increase rate of [Ca^2+^]_*i*_ and *V*_*m*_ should be two groups of principal parameters to evaluate the effects of rogue RyRs on Ca^2+^ dynamics and electrophysiological properties. Besides Δ[Ca^2+^]_*i*_ and Δ*V*_*m*_, two new parameters *T*_peakCa_ and *T*_peak*V*_*m*__ are used in our simulation. *T*_peakCa_ represents the mean time to reach the peak of [Ca^2+^]_*i*_ from the end of resting stage, and *T*_peak*V*_*m*__ is the time to reach the peak of *V*_*m*_. Increase rate of Ca^2+^ concentration and depolarization velocity could be estimated indirectly by the two parameters, which is shown in Figure 3(b). The simulated *T*_peakCa_ decreases significantly when *N*_rogue_ is upregulated from 0 to 0.25 rogue RyR/*μ*m^2^ (*T*_peakCa_ = 418 ± 19.7 ms and 325 ± 15.2 ms (*n* = 10), resp., *n* = 10, *P* < 0.05). However, decrease of *T*_peak*V*_*m*__ is slight when *N*_rogue_ is from 0 to 0.25 rogue RyR/*μ*m^2^ (*T*_peak*V*_*m*__ = 406 ± 38.5 ms and 340 ± 25.1 ms, resp., *n* = 10, *P* > 0.05). In Figure 3(c) three blue curves when Time >1000 ms represent repeated simulation results of *V*_*m*_ without any stimulus when *N*_rogue_ = 0.25 rogue RyR/*μ*m^2^. The results indicate a smooth variation without a significant peak in the membrane potential morphology.

When *N*_rogue_ is increased to 0.5 rogue RyR/*μ*m^2^, similar to above, small spontaneous calcium waves cannot propagate in a long distance and quickly decay as well. The depolarization of membrane potential caused by calcium release events has bigger amplitude compared with that when *N*_rogue_ = 0.25 rogue RyR/*μ*m^2^ (*P* < 0.05), but is also relatively weak with an average of 7.48 ± 0.25 mV. Again, the membrane potential morphology is smooth.

**Figure 4:**
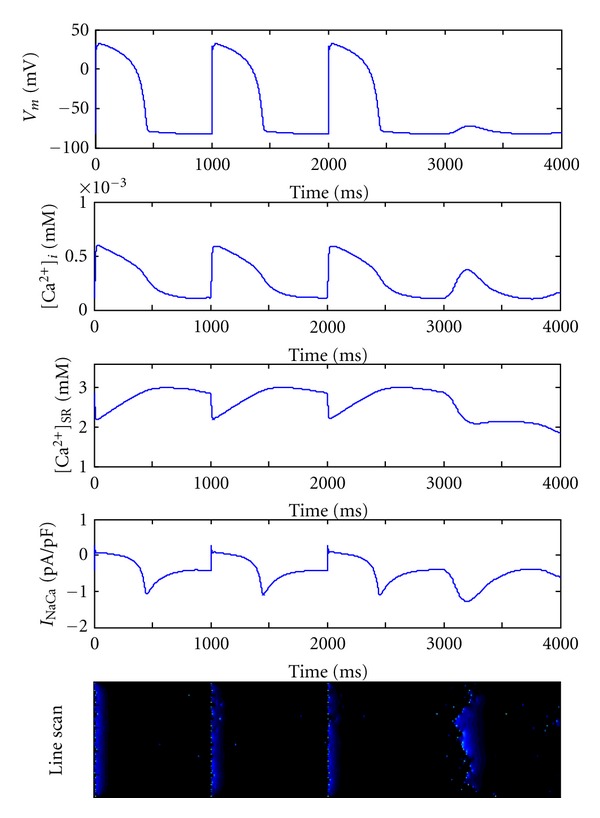
Occurrence of DAD following three action potentials in failing myocyte when *N*_rogue_ = 1.0 rogue RyR/*μ*m^2^. Five figures from top to bottom represent membrane potential, cytoplasmic [Ca^2+^]_*i*_, SR luminal [Ca^2+^]_SR_, *I*_NaCa_, and line-scan image along longitudinal direction of the cell, respectively, in failing ventricular myocyte.

 However, as *N*_rogue_ is further increased, specifically when *N*_rogue_ ≥ 0.75 rogue RyR/*μ*m^2^, large calcium waves could be initiated spontaneously in the 2D subcellular region. Moreover, our Monte-Carlo simulation results show that these large calcium release events cause a significant larger calcium transient at the whole cell level and subsequently depolarize the membrane potential to a larger extent when *N*_rogue_ is increased by a step of 0.25 rogue RyR/*μ*m^2^ (*P* < 0.05) as shown in Figure 3.  Figure 4 shows typical simulation results when *N*_rogue_ = 1.0 rogue RyR/*μ*m^2^. After 3 action potentials paced by the cycle length of 1000 ms, a relatively large Ca^2+^ transient is observed, as well as a big inward *I*_NaCa_ and consequently a DAD without external stimulus in the heart failure myocytes. The linescan image in Figure 4 indicates the underlying microcosmic Ca^2+^ cycling on the subcellular level.

Besides, as *N*_rogue_ is increased to a larger value, Ca^2+^ transient elicited by spontaneous Ca^2+^ release and the depolarization of membrane potential enlarge further (*P* < 0.05), while the values of *T*_peakCa_ and *T*_peak*V*_*m*__ decrease significantly (*P* < 0.05) (as shown in Figure 3). All these results together suggest close relationship between rogue RyRs and DADs.

### 3.3. Triggered Action Potential

As demonstrated above, the spontaneous Ca^2+^ release from SR causes a Ca^2+^ transient in cytoplasm and subsequently depolarizes the membrane potential even in the resting stage without any stimulus. Furthermore, the amplitude of calcium transient and the degree of depolarization are positively correlated to the distribution density of rogue RyR channels. Therefore, once the density of rogue RyRs is large enough, the membrane potential may be depolarized to reach the threshold that will trigger an action potential.

**Figure 5:**
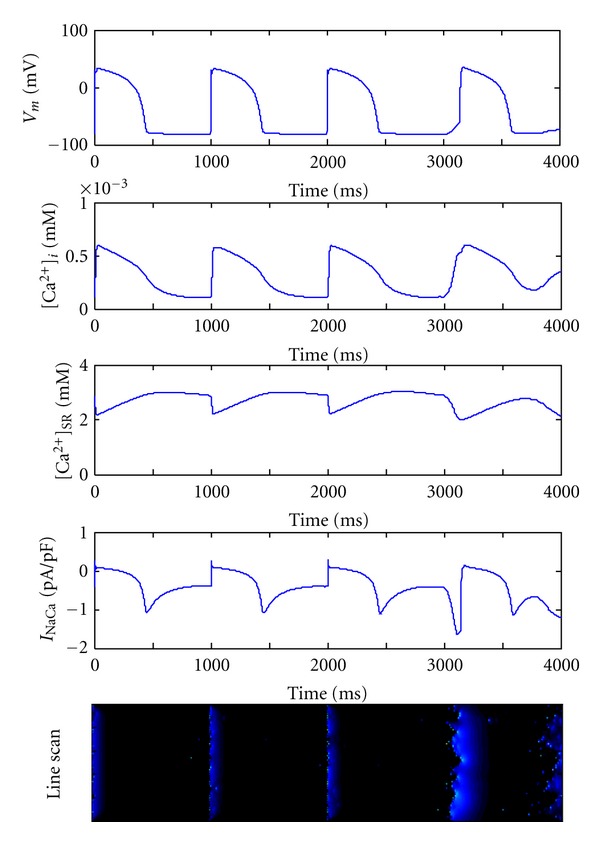
Triggered action potential in failing myocyte when *N*_rogue_ = 1.75 rogue RyR/*μ*m^2^. Five figures from top to bottom represent membrane potential, cytoplasmic [Ca^2+^]_*i*_, SR luminal [Ca^2+^]_SR_, *I*_NaCa_, and line-scan image along longitudinal direction of the myocyte, respectively, in failing ventricular cell.

Figure 5 shows the time course of simulated membrane potentials: cytoplasmic Ca^2+^ concentration, SR Ca^2+^ store, the Na/Ca exchange current, as well as the Ca^2+^ dynamics at subcellular level. From the line-scan image we can see that, along the cellular longitudinal direction, many spontaneous Ca^2+^ waves are initiated nearly at the same time. These Ca^2+^ waves could propagate and diffuse to finally form a large wave. These Ca^2+^ releases rise the [Ca^2+^]_*i*_ quickly and drive a strong inward component of *I*_NaCa_, which causes the depolarization of the membrane potential. When the membrane potential is depolarized to reach the threshold for activation of fast Na^+^ channel, the large *I*_Na_ is produced very fast and induces an upstroke of the membrane potential. Then, the L-type Ca^2+^ channels will be subsequently activated and a flux of extracellular calcium ions burst into cytoplasm via the L-type calcium channels. This inflow of Ca^2+^ together with the previously released Ca^2+^ from SR can activate the remaining available RyR channels and elicit an even larger Ca^2+^ transient in the cytoplasm. The other ionic channels at the membrane are successively opened and determine the morphology of AP together. Particularly, for the *I*_NaCa_, it switches to a weak outward current during the plateau stage, but turns to a strong inward current in the repolarization stage of AP, by which the Ca^2+^ is ejected to the extracellular space.

 When *N*_rogue_ = 1.5 rogue RyR/*μ*m^2^, triggered APs are observed in 11 simulations (totally 22 Monte Carlo simulations), that is, the probability of triggered AP is 50%. Moreover, when *N*_rogue_ = 1.75 rogue RyR/*μ*m^2^, triggered APs are found in 18 out of 21 Monte Carlo simulations, that is, the probability of triggered AP rises to 87.5%. On the contrary, when *N*_rogue_ < 1.5 rogue RyR/*μ*m^2^, no triggered AP is seen in our simulations.

To quantitatively investigate the effect of high dense rogue RyRs on Ca^2+^ handling, we also recorded the variations of [Ca^2+^]_*i*_ and membrane potential (Δ[Ca^2+^]_*i*_ and Δ*V*_*m*_) as well as *T*_peakCa_ and *T*_peak*V*_*m*__. However, Δ*V*_*m*_ elicited by intensive Ca^2+^ release events is often large enough to activate *I*_Na_. Consequently, the voltage upstroke induced by *I*_Na_ would overlap the original Δ*V*_*m*_. Then, Δ[Ca^2+^]_*i*_ by Ca^2+^ release events is also overlapped by the following inward-flowing *I*_CaL_ during a triggered AP. Under those conditions, the measurement of Δ[Ca^2+^]_*i*_ and Δ*V*_*m*_ as well as *T*_peakCa_ and *T*_peak*V*_*m*__ becomes very difficult. Thus, in our simulation, when *N*_rogue_⩾ 1.5 rogue RyR/*μ*m^2^, we set the *I*_Na_ to be zero after removing the external stimulus. By doing this, triggered AP will not be formed even when a DAD makes the membrane potential more positive than the threshold for *I*_Na_. Therefore, we can calculate these parameters easily. Actually, our simulation results are shown in Figure 3 marked by arrows, from which we can conclude that, as *N*_rogue_ is increased from 1.5 to 1.75 rogue RyR/*μ*m^2^, the amplitude of [Ca^2+^]_i_ enlarges (*P* < 0.05), whereas the time to reach the peak does not change obviously (*P* > 0.05). However, the amplitude of a DAD increases significantly (*P* < 0.05), while the time to observe the DAD decreases (*P* < 0.05).

## 4. Discussion

### 4.1. Mechanism of Ca Handling in HF

Heart failure, a syndrome caused by significant impairments in cardiac function, has become one of the biggest human killers with a poor prognosis [30]. Ca^2+^ handling of cardiac cells in heart failure is always characterized by reduction in the amplitude as well as by slowed decay of Ca^2+^ transient [31]. The primary reason for decrease in the amplitude of Ca^2+^ transient is the partly unloaded Ca^2+^ store in SR. Three factors mainly accounting for the smaller store are (1) increased Ca^2+^ leak in the resting myocyte, (2) decreased activity of SR Ca^2+^ pump, and (3) increase in expression and/or activity of Na^+^-Ca^2+^ exchanger. 

Despite the increased activity of Na/Ca exchanger, at the early stage of [Ca^2+^]_*i*_ decay, the net current of Na/Ca exchanger might be outward current (i.e., Ca^2+^ influx) or weak inward current, due to longer AP plateau and higher plateau potential in failing myocyte. Therefore, slowed decay of Ca^2+^ transient is mainly due to decreased SR Ca^2+^ pump, which removes major amount of Ca^2+^ at the early stage of [Ca^2+^]_*i*_ decay. Only when the membrane potential is repolarized to a relatively negative voltage and [Ca^2+^]_*i*_ is still high that *I*_NaCa_ turns to a strong inward current and accelerates decay of Ca^2+^ transient at the late stage of [Ca^2+^]_*i*_ decay.

### 4.2. Arrhythmogenic Effect of Rogue RyRs

Besides pump failure, patients with severe heart failure are at high risk of sudden cardiac death generally triggered by a lethal arrhythmia [23, 32]. DADs are thought to be the primary mechanism underlying arrhythmia in failing heart [33]. In our simulation work, although the probability of firing of RyR clusters increases in resting failing cardiocytes, the spontaneous Ca^2+^ sparks could not elicit enough amplitude of Ca^2+^ transient to induce an obvious DAD in the absent of rogue RyRs. The existence of rogue RyR channels is of importance in initiation and propagation of spontaneous Ca^2+^ waves in ventricular myocytes with heart failure [14]. 

In this work, we propose a coupled mathematic model by integrating the spatiotemporal Ca^2+^ reaction-diffusion system into the cellular electrophysiological model. Rogue RyR channels are then incorporated into the coupled model to simulate subcellular Ca^2+^ dynamics and global cellular electrophysiology simultaneously under heart failure conditions. Our simulation results show that, in the presence of rogue RyRs, Ca^2+^ dynamics is more unstable and Ca^2+^ waves are more likely to be initiated than the condition without rogue RyRs. Different from sporadic sparks in a resting myocyte without Ca^2+^ waves, a number of SR Ca^2+^ release events occur intensively during the process of spontaneous occurrence of Ca^2+^ waves. These release events could elevate the amplitude of Ca^2+^ transient effectively and thus induce Ca^2+^-dependent inward current (mainly via Na/Ca exchanger) which depolarizes the sarcolemma and lends to a DAD, or a triggered AP sometimes. For a given level of Ca^2+^ release in failing myocytes, inward depolarizing current becomes larger due to increased activity of Na/Ca exchanger. And increased membrane resistance owing to reduction of *I*_K1_ enables the same inward current to produce greater depolarization. Once a DAD elevates membrane potential to the threshold for activation of *I*_Na_, a triggered AP is then formed. DADs and triggered AP are the primary triggered activities accounting for arrhythmias in heart failure. 

### 4.3. Dependence of *V*_*m*_ on Density of Rogue RyRs

Without rogue RyR channels or with low distribution density, occurrence of spontaneous Ca^2+^ sparks and/or quarks is independent in time and space, which is unlikely to evolve into propagating Ca^2+^ waves with partially unloaded SR Ca^2+^ store. [Ca^2+^]_*i*_ variation elicited by these Ca^2+^ release events is slight. In our simulation, when *N*_rogue_ = 0 or 0.25 rogue RyR/*μ*m^2^, the amplitude of membrane potential depolarization is very small, only several mV, and the average *T*_peak*V*_*m*__ is big and extensive with large SEM. The morphology of membrane potential is very smooth without a distinct peak, so that this type of depolarization could not be referred to a genuine DAD. 

However, as more rogue RyRs are distributed over the 2D plane, Δ*V*_*m*_ increases gradually, while the value of *T*_peak*V*_*m*__ decreases but becomes more intensive. When the value of *N*_rogue_ is elevated to 1.5 rogue RyR/*μ*m^2^ or bigger, the large membrane potential depolarization tends to evoke a triggered AP, and the probability of occurrence of triggered APs increases with the larger *N*_rogue_. The reason is that larger number of rogue RyRs would increase the amplitude and rate of DADs by initiating more Ca^2+^ waves which occur more synchronously. Therefore, depolarization of *V*_*m*_ is indicated to be dependent on the distribution density of rogue RyR channels. 

### 4.4. Limitations and Further Work

Because the rogue RyR remains to be a hypothetical channel rather than a determinate concept, experimental parameters of rogue RyRs are lacked. Some assumptions were made regarding the density, distribution, and kinetic of rogue RyR channels. In our work, a constant was used to represent Ca^2+^ release flux via a rogue RyR channel, while Ca^2+^ release flux via a cluster of RyRs was dependent on global luminal Ca^2+^ concentration ([Ca^2+^]_SR_) and local Ca^2+^ concentration ([Ca^2+^]_*i*(*xi*,*yi*)_). Besides, different *N*_rogue_ values were used to evaluate the effect of rogue RyR on Ca^2+^ cycling and membrane potential in failing heart.

Numerous key regulatory proteins, such as protein kinase A(PKA), Calstalin, CaMKII, and phosphatase, are bound to RyR, thus forming the junctional complex. RyR channels would be regulated via different signaling pathways [34]. For example, PKA phosphorylation dissociates FKBP12.6 from the RyR and thus makes the RyR channel unstable in failing hearts [35]. These regulating processes could not be mimiced by our present model. Besides, defective Ca^2+^ handling also occurs in many cardiac diseases, such as myocardial infarction, atrial fibrillation, and various arrhythmogenic paradigms. The coupled model is planned to be improved, and more relevant parameters should be added to investigate the potential mechanisms of Ca^2+^ dynamics in various kinds of cardiac diseases.

## 5. Summary

By integrating the spatiotemporal Ca^2+^ reaction-diffusion model into the cellular electrophysiological model, appearance of subcellular Ca^2+^ release events and evolution of waves together with dynamics of ionic concentration and membrane potential on the cellular level could been monitored simultaneously. By using the coupled model we investigate the effects of rogue RyRs on Ca^2+^ handling from subcellular to cellular level as well as electrophysiological properties in failing heart. The simulation results suggest that rogue RyR with tiny Ca^2+^ release flux should be an important factor in triggering arrhythmia in failing cardiac cells. Our work suggests the importance of rogue RyRs in initiation of Ca^2+^ release events (especially Ca^2+^ waves) and consequently DADs or triggered APs. Our study indicates the arrhythmogenic effect of rogue RyRs and helps to elucidate a possible arrhythmia mechanism in failing heart.

## Figures and Tables

**Table 1:** Parameters in nonfailing versus failing myocyte models.

Parameters	Definition	Nonfailing	Failing
*V*_max⁡_	Maximal SR Ca^2+^ pumping rate	0.006375 mM/ms	0.0035 mM/ms
*G*_K*s*_	Maximal *I*_Ks_ conductance	0.392 nS/pF	0.196 nS/pF
*G*_K1_	Maximal *I*_K1_ conductance	5.405 nS/pF	4.324 nS/pF
*G*_to_	Maximal *I*_to_ conductance	0.294 nS/pF	0.185 nS/pF
*G*_Na_	Maximal *I*_Na_ conductance	14.838 nS/pF	8.902 nS/pF
*P*_NaK_	Maximal *I*_NaK_	2.724 pA/pF	1.57 pA/pF
*G*_bCa_	Maximal *I*_bCa_ conductance	0.000592 nS/pF	0.0009045 nS/pF
*k*_NaCa_	Maximal *I*_NaCa_	1000 pA/pF	1650 pA/pF
*K*_*m*_	Ca^2+^ sensitivity parameters of RyR clusters or rogue RyR when they take the same value	15 *μ*M	7.5 *μ*M
*D*_SR_	luminal Ca^2+^ sensitivity parameter of Ca^2+^ release events	3.25 mM	2.5 mM
